# Comparison and quantitative analysis of microstructure parameters between original loess and remoulded loess under different wetting-drying cycles

**DOI:** 10.1038/s41598-020-62571-1

**Published:** 2020-03-26

**Authors:** Wan-kui Ni, Kang-ze Yuan, Xiang-fei Lü, Zhi-hui Yuan

**Affiliations:** 1 Department of Geological Engineering, College of Geological Engineering and Surveying and Mapping, Chang’an University, No.126 Yanta Road, Xi’an, Shaanxi 710054 P.R. China; 2 School of Water and Environment, Chang’an University, No. 126 Yanta Road, Xi’an, Shaanxi 710054 P.R. China; 3 College of Water Conservancy and Ecological Engineering, Nan Chang Institute of Technology, Nan Chang, Jiangxi 330099 P.R. China

**Keywords:** Geochemistry, Geology, Structural geology, Geomorphology

## Abstract

The microstructural evolution of loess had a significant impact on the collapsibility of loess during wetting-drying cycles. Based on the analysis of scanning electron microscope (SEM) images by using Image-Pro Plus, the present study quantitatively compared the microstructural parameters of original loess and remoulded loess with different moisture content before and after wetting-drying cycles in size, shape, and arrangement. In size, the average diameter of both original loess particles and remoulded loess particles increased with the increasing of initial moisture content. However, the average diameter of original loess particles was slightly larger than that of remoulded loess particles before wetting-drying cycles. In contrast, the average diameter of both original loess particles and remoulded loess particles were very close to each other after three wetting-drying cycles. In shape, before wetting-drying cycles, the average shape factor of original loess particles was higher than that of remoulded loess particles. After three wetting-drying cycles, the difference in the average shape factor of both two loess samples with 5% initial moisture content is similar to that before wetting-drying cycles. Nevertheless, the average shape factor of both original loess particles and remouled loess particles with 15% initial moisture content were very close to that with 25% initial moisture content. In the arrangement, directional frequency indicated remoulded loess appeared to be more vertically aligned than original before and after three wetting-drying cycles. Furthermore, the directed anisotropy rate of remoulded loess was higher than that of the original loess before and after three wetting-drying cycles. In summary, the size, shape, and arrangement of both original loess particles and remoulded loess particles varied in different degrees before and after three wetting-drying cycles. Combined with the water retention curve of the loess, we analyzed the microstructural evolution mechanism of two loess particles during wetting-drying cycles. It is an excellent significance to study the engineering properties of original loess and remoulded loess.

## Introduction

As widespread continental sediment, loess covers about 10% of the total land area of the world. At the same time, China exhibits the most extensive distribution in the loess region of the world, especially for Chinese loess plateau, with a total area of about 640,000 km^2^. In China, loess deposits account for more than 6% of the territory and are mainly distributed in the regions of Shanxi, Shaanxi, Gansu, and Ningxia^1^. Shallow loess (Q_3_ loess) in the Loess Plateau over northwestern china is typical aeolian loess, which is mainly composed of coarse powders, including a fraction of clay minerals, soluble salts, and CaCO_3_. Therefore, loess soil has a metastable structure with aggregates and bracket macropores^2,3^. The main engineering-geological problem of this loess is significantly reduced strength and large collapsing deformation under wet stress path and external load.

Increasing amounts of research have been performed on the microstructure of loess to investigate its tendency to collapse and its engineering properties^4,5^. Since the 1960s, studies on the microstructure of loess have changed from the initial qualitative analysis approach to the current quantitative analysis. These quantitative analyses mainly contain different calculated or statistical parameters based on the many methods of characterizing loess and have included the use of microscopes, electron microscopes, computer tomography (CT) scanners, mercury intrusion porosimetry (MIP), Brunauer Emmett Teller measurement (BET) and scanning electron microscopy (SEM)^6–9^. Among these characterization techniques, SEM has become one of the most common tools in researching the microstructure of loess. SEM images show the aggregation of particles, and also helps to determine the morphology of soil pores. All this information is essential for predicting the likelihood of unsaturated loess collapsing^10,11^.

Based on SEM images, recent research on the microstructure of loess has made some progress. Xie *et al*.^12^ described the microstructural evolution and quantitatively compared the pore areas, effective circle diameter, orientation angle, and shape ratio of loess samples by processing the SEM images using Image-Pro Plus (IPP) software before and after the collapse. Depending on these results, they suggested that the contact between particles transformed from a point-contact to a face-contact type of relationship under loading and wetting conditions. Li *et al*.^13^ used the soil pore parameters (including their area, major axis length, eccentricity, and orientation) determined from SEM images to explain the microstructural evolution and mechanical responses of two loess soils in various tests. These research papers are valuable for their investigation of pore parameters due to their quantitative microstructure data and for understanding the interaction between the mechanical behavior and pore parameters of loess.

In the past two decades, some researchers have carried out on water retention and mechanical properties of loess under wetting-drying cycles, based on the mechanical theory of unsaturated soil. At the same time, a series of laboratory experiments were used to observe the evolution of loess porosity under wetting-drying cycles or external loading^14–16^. However, the main factor which influences the mechanical behavior of unsaturated loess is the fabric of loess, including aggregates and cemented structure, the quantitative parameters of pore morphology properties cannot be used to describe the microstructural evolution of loess particles and aggregates completely. Therefore, how the loess particles’ microstructure evolves due to different wetting-drying cycles still requires further investigation. Besides, the strength and deformation characteristics of original loess exhibited different from that of remoulded loess under the same density and water content. Remoulded loess has commonly been used to replace the original loess in practical engineering due to reducing collapsibility. Because remoulded loess exhibited a similar chemical composition and particle distribution as the original loess. As a result, the microstructural variations of two loess samples were the main reason causing different engineering mechanical performances between original loess and remoulded loess in actual engineering. However, whether the remoulded loess shares some of the microstructural properties of the original loess is still unknown. The comparisons of the microstructural variations between original loess and remoulded loess under different wetting-drying cycles are rare in the literature.

It is a significant work to distinguish the difference in particles’ microstructure between original loess and remoulded loess. Especially, how does the microstructure of two loess particles lead to the variety in mechanical properties? After wetting-drying cycles, original loess exhibited porous structure and collapsibility, while the remoulded loess displayed reduced collapsibility and increasing compression resistance. Why did they demonstrate such difference, even in the same density and moisture content?

In light of these considerations, this paper explores the quantitative microstructural parameters to analyze the size, shape, and arrangement of original loess and remoulded loess particles resulting under different wetting-drying cycles, based on processing SEM images using IPP. The new findings of this work are considerable significance to study the engineering properties of original loess and remoulded loess.

## Materials and Methods

### Loess samples

**Figure 1:**
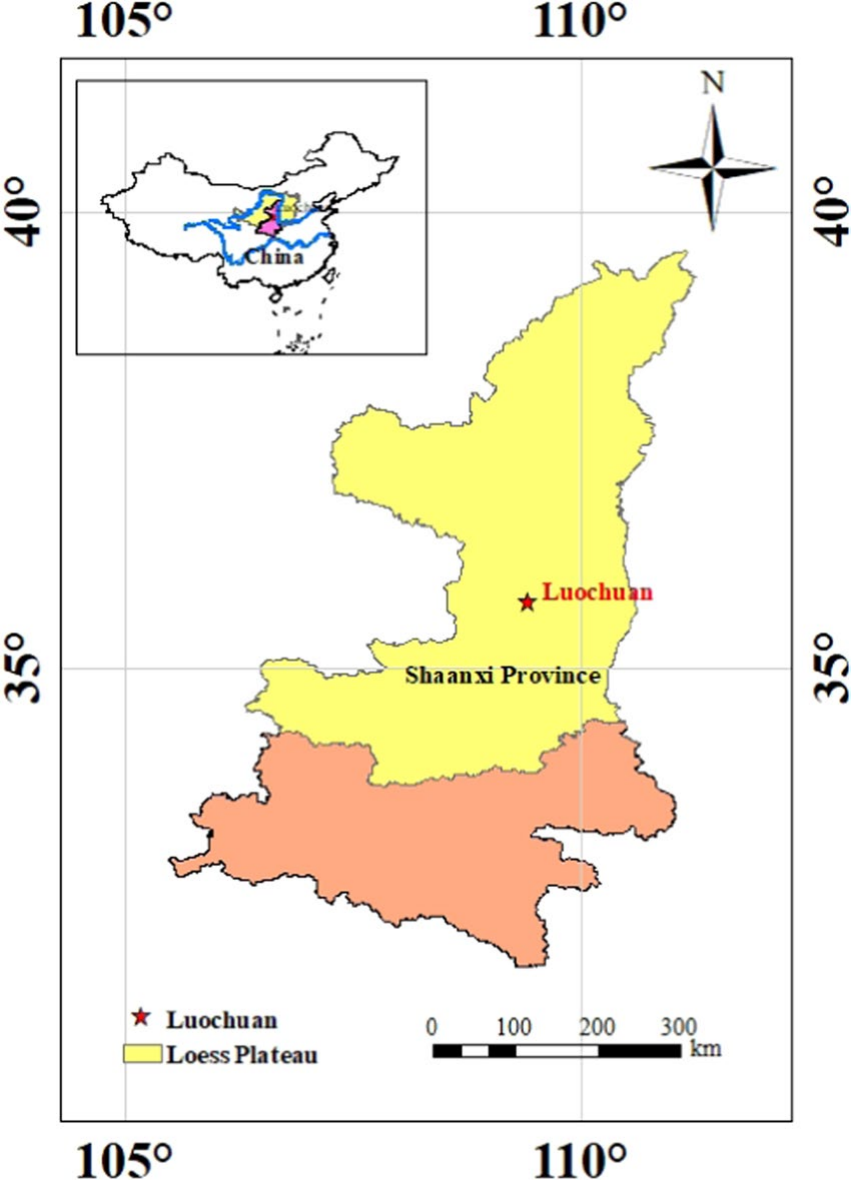
The Major occurrence of Loess Plateau in China and sampling location at excavation site (latitude 35° 42′46.09′′N, longitude 109°26′0.10′′E).

**Figure 2:**
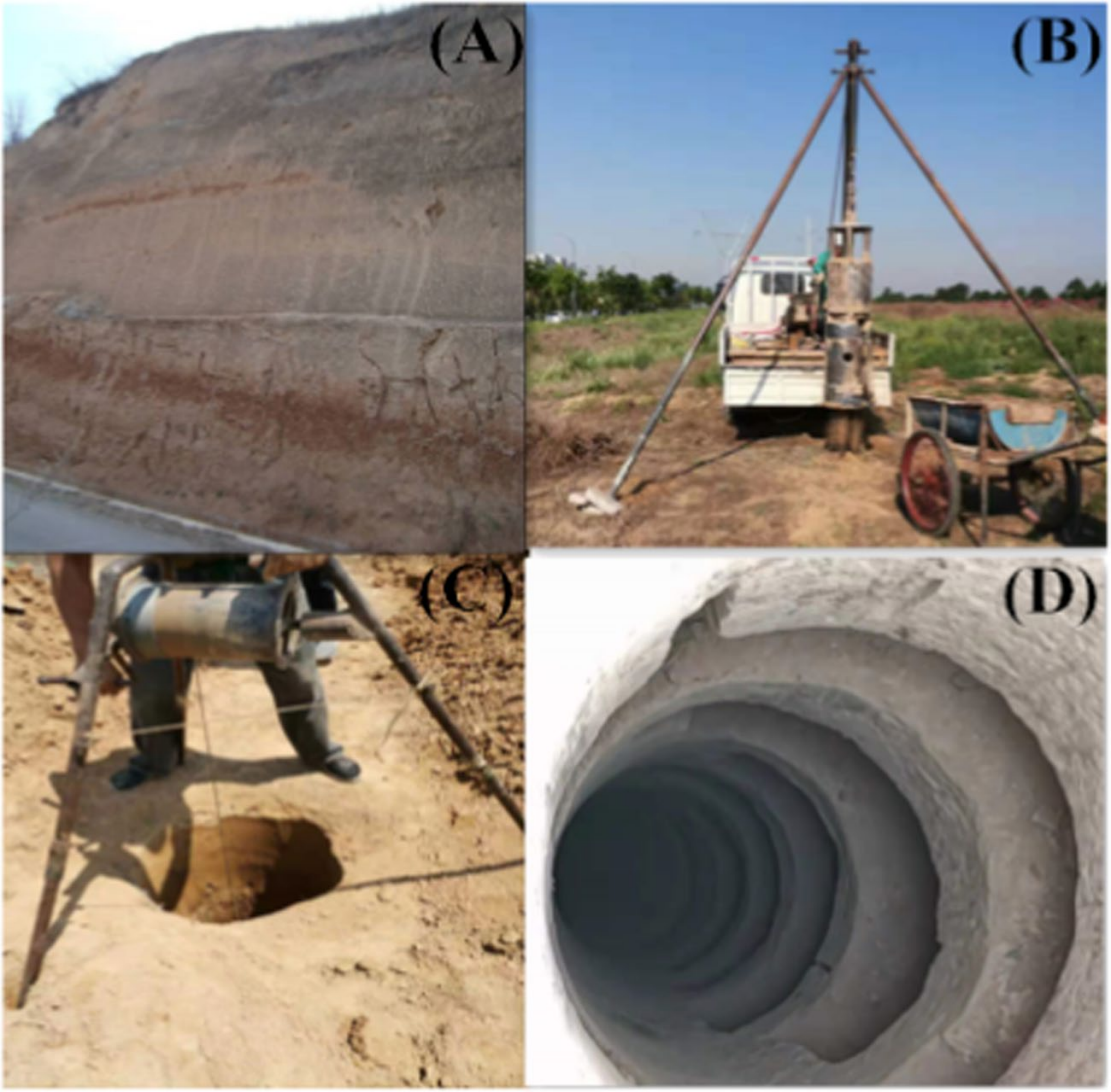
Sample collection. (**A**) a view of sampling site; (**B**) sampling equipment; (**C**) sampling process; (**D**) trial pits.

Undisturbed loess samples were collected from the Loess Plateau in Shaanxi Luochuan Loess National Geopark (35°42′46.09′′N, 109°26′0.10′′E), China. Figure 1 shows the major distribution map of the Loess Plateau in China and sampling location. The trial pits were pumped to collect original loess samples at depths ranging from 2.5 m to 4.0 meters below the ground surface. In order to minimize changes to their properties, all the samples were retrieved by hand, sealed in the plastic film inside a cylindrical iron bucket, and transported to the laboratory. A photo of the trial pits is exhibited in Fig. 2. The tested loess samples were light brown silty soil with slight plasticity. The main physical properties of the loess were determined following the ASTM 2006 standard test methods^17^, as listed in Table 1.

**Table 1:** Basic characteristics of loess test samples.

Sample measure-ments	*In situ* density(g/cm^3^)	Natural moisture content (%)	Specific gravity	plastic limit(*ω*_*P*_/%)	Liquid limit(*ω*_*L*_/%)	Plasticity index (*I*_*p*_)	void ratio
Loess	1.18~1.22	11	2.72	19.5	28.8	9.3	1.24

### Preparation of loess samples

After collection, the original loess samples were extracted as a cylindrical clod with the aid of a greased ring 40 mm in diameter and 100 mm in height. To achieve the desired moisture level, we gradually dropped water into the samples using a dropper. Then the wetted loess samples were sealed in black polyethylene bags for 72 hours in order to reach moisture equilibrium.

The remoulded loess samples were prepared as follows. First, the collected loess was passed through a 2 mm sieve, then it was oven-dried at 110 °C for 8 hours and naturally cooled to room temperature. Then the amount of deionized water calculated from Eq. (1) was gradually added to the dry loess particles using a spray bottle until the moisture content was up to the requirement. We followed the same steps with the wetted loess samples to achieve the same moisture equilibrium used with the original loess. Finally, the samples were compacted using the floating ring mold directly in the oedometer cylinder (producing a final sample diameter of 40 mm and height of 100 mm). The remoulded samples were compacted to the same initial conditions as the original loess.

Deionized water was used to wet the loess clod until the loess reached the required moisture content. For all the samples, the amount of water added was determined using the following equation:

1 $${m}_{w}=\frac{0.01\times (w-{w}_{0})}{1+0.01{w}_{0}}\times {m}_{0}$$

where *m*_*w*_ is the final water content of the sample, *m*_0_ is the initial moisture content of the sample, *w* is the weight of the added water, and *w*_0_ is the original weight of the water in the sample. The moisture content of the remoulded sample was controlled using the same process.

### Wetting-drying cycle

**Figure 3:**
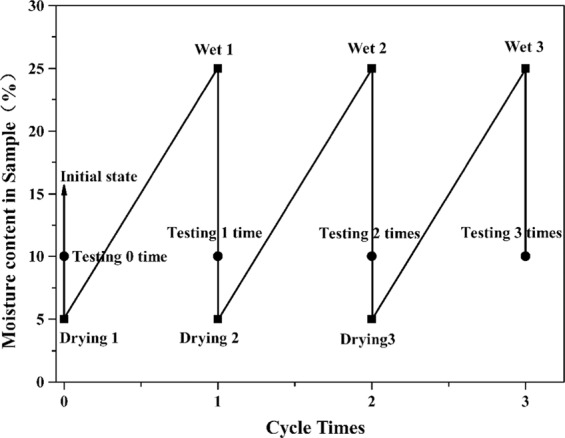
Wetting-drying cycle testing process.

We measured the water content of the loess at the sampling site and found that the water content ranged from 5% to 25%. Chen Rui *et al*. has witnessed three wetting-drying cycles that caused the loess samples’ changes in microstructure and mechanical behavior^18^. Therefore, the samples in this study underwent different wetting-drying processes as follows: first, we chose the prepared samples in section 2.2 with three initial moisture content and air-dried to 5% water content. Then, the samples were wetted to reach the 25% moisture content level by dripping water at the bottom. They wrapped carefully and strictly using plastic film and placed into the humidor for homogenized in room temperature. This comprised one wetting-drying cycle. Finally, we subjected the samples to the wetting-drying cycle circles three times. The described process is illustrated in Fig. 3.

### Microstructure investigations

Cubic sticks, having the dimensions of approximately 1 cm × 1 cm × 2 cm (length × width × height, were trimmed out from the central part of the loess samples after the wetting-drying cycles. Before scanning, the soil sticks were slightly fractured by hand at about 1 cm height, and the new surface was used to examine the microstructure of the samples. Figure S1 shows the surface of the samples by the SEM. Then, half the stick was stuck to the shooting pad for sputter coating with platinum (Pt) in sputtering ion equipment, using electron-conductive tape without disturbing the fractured plane. A Quanta 200FEG SEM was used to record the microstructure photos of all samples. All the images have been included in the Supporting Information (Supp. Info Figs. S2–S5).

### Data collection

**Figure 4:**
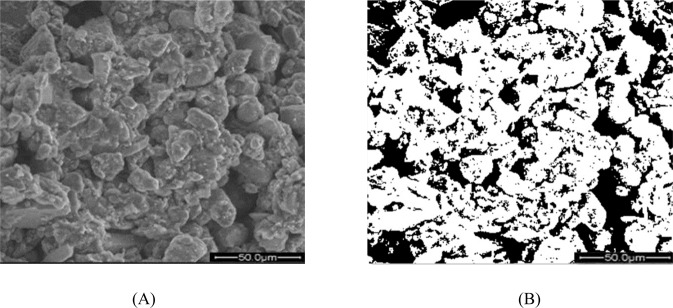
Micrographs of remoulded particles with 25% moisture content after three wetting-drying cycles: (**A**) overall view; (**B**) binarized view (white-colored areas are particles, and black areas are pores).

To quantify the microstructural characteristics of the samples, statistical analysis was conducted on the morphology properties of loess particles. The IPP software for image processing was used to distinguish the soil particles and pores. The SEM micrographs, magnified 1000-fold, were processed to provide useful information about the particle distribution in the soil structure. The quantitative processing was carried out as follows. Firstly, the micrographs were imported into the IPP software as binarized images (shown in Fig. 4). By examining the binarized images, it was easy to separate the loess particles and pores from the soil shown in the images. Then the core of binarization was the selection of an image threshold. We changed the grey value of each image and took the images three times to calculate the standard deviation. Then, the following parameters were divided to three types: size (the average diameter ***d***), shape (the ratio of short diameter to long diameter ***C****,* circularity ***R***, the average shape factor ***F***, and morphological fractal dimension ***D***) and arrangement (Directional frequency ***F(α)***, anisotropy rate ***I***_***n***_, directional probability entropy ***H***_***m***_, and directional fractal dimension ***D***_***f***_). All these parameters were chosen as the microstructural parameters of the sample loess particles^19,20^. The parameters were calculated as follows:

The average diameter ***d*** is the diameter of the equivalent circle, whose area is equal to the area of the loess particle so that the size of the particle can be described by Eq. (2).

2 $${\rm{d}}=\sqrt{\frac{4S}{\pi }}$$

where *S* is the area of the equivalent circle.

The ratio of short diameter to long diameter (***C***) is used to describe the sharp of loess particle. A large value of ***C*** indicated that length of the loess particle is similar to the width of the loess particle, and the sharp of loess particle tends to round or square in-plane, while a small of value ***C*** illustrated great gap between length and width and the loess particles inclined to the rectangular or oval shape. It can be calculated by Eq. (3).

3 $${\rm{C}}=\frac{B}{L}$$

where *L* is the length of the long axis of the loess particle, and *B* is the width of the short axis of the loess particle. ***C*** is the ratio of B to L, which fluctuated from 0 to 1.

Next, we applied the average shape factor (*F*) to reflect the variation in the shape of the loess particles. It can be calculated by Eq. (4).

4 $${\rm{F}}=\frac{{\sum }_{i=1}^{n}F}{n}\,({F}_{i}=P/S)$$

where P is the perimeter of the equivalent circle, S is the actual perimeter of the loess particles, *F*_*i*_ is the shape factor of the loess particles, and n is the total number of selected particles in the SEM images.

Furthermore, we applied Eq. (5) to obtain the value of circularity ***R***, which can accurately estimate how similar the loess particle in SEM is to the equivalent circle.

5 $${\rm{R}}=\frac{A}{A{\prime} }$$

where A is the actual area of the loess particle, and A′ is the circumcircle area of the loess particle.

The directional fractal dimension ***D****,* proposed by Moore and Donaldson (1995), was used to analyze the surface characteristics of loess soil and their influence on macroscopic strength. It can be calculated as following Eq. (6):

6 $$\log (Perimeter)=\frac{D}{2}\times \,\log (Area)+C$$

where the *Perimeter* and *Area* are the perimeter and area of the equivalent circle, respectively, ***D*** is the morphological fractal dimension of the loess particle, and ***C*** is the fitting constant.

The strength of intact, homogeneous loess will be significantly influenced by the particle arrangements at the microscale level. Directional frequency ***F(α)***, anisotropy rate ***I***_***n***_, directional probability entropy ***H***_***m***_, directional fractal dimension ***D***_***f***_ were used to investigate the arrangement characteristics of loess particles, which can be used to reveal the changes of these particles during a deformation process.

The directional frequency (*F(α)*), indicated how significantly the particles were aligned in a specific direction. Firstly, we calculated it by using the directional angle, which can be described as the angle between the length of particles and the X-axis. Secondly, the variable directional angle was divided 180° into nine angle intervals, where the range of angles is 20° (Δ*θ* = 20°). Finally, *F(α)* can be defined as the frequency of particles that fall into each interval [θ_i_, θ_i+1_], as Eq. 7.

7 $${\rm{F}}(\alpha )=\frac{{n}_{\alpha }}{n}$$

where *n*_*α*_ is the number of loess particles whose directional angle changed from *θ*_*i*_ to *θ*_*i+1*_, and n is the total number of loess particles in the SEM image.

The anisotropy rate *I*_*n*_ is a quantitative parameter, which can reflect the arrangement of loess particles. It is defined by Eq. (8). The value of anisotropy rate alters from 0 to 1. When the value of *I*_*n*_ is 0, the orientation of loess particles is completely isotropic arrangement. Conversely, when the value of *I*_*n*_ is 1, the loess particles exhibit chaotic distribution.

8 $${I}_{n}=\frac{L-B}{L}\times 100 \% $$

where L is the length of the long axis of the loess particle, and B is the width of the short axis of the loess particle.

We then performed the Eq. (9) to obtain the directional probability entropy (*H*_*m*_). It normally used to describe the directionality and arrangement of loess particles. The closer to 1 the value of *H*_*m*_ is, the more randomly distributed the loess particles were, and vice versa.

9 $${H}_{m}=-\,\mathop{\sum }\limits_{i=1}^{n}{F}_{i}(\alpha ){\log }_{n}{F}_{i}(\alpha )$$

where *F*_*i*_*(α)* is the directional frequency. The value of *H*_*m*_ changed from 0 to 1.

Our next calculation was the directional fractal dimension (*D*_*f*_) *by* the Eq. (10). *D*_*f*_ is the slope of the linear relationship between $\mathop{\sum }\limits_{i=1}^{n}{F}_{i}(\alpha )\mathrm{ln}({F}_{i}(\alpha )$ and lnα. The lower the *D*_*f*_ value is, the more neatly particles are arranged.

10 $${D}_{f}=\frac{{\sum }_{i=1}^{n}{F}_{i(\alpha )\mathrm{ln}({F}_{i}(\alpha ))}}{\mathrm{ln}\,\alpha }$$

where *F*_*i*_*(α)* is the directional frequency, *α* is the directional angle.

## Results and Discussion

### The size of loess particles

The average diameter (***d***) of different particles in the original loess and remoulded loess were used to illustrate the size of loess particles, and listed in Table 2. The ASTM standards and Zhong-xiu Sun *et al*. reported that the sizes of the loess structural units were below 100 μm, and the particles with a diameter of larger than 100 μm were considered to the loess aggregates^21,22^. We determined that the loess particle with an average diameter below 100 μm was the applicable scale range for this work. It can be seen from Table 2 that the average diameter of the loess particles varied from 18.27 μm to 27.97 μm. The average diameter of the particles (both the original loess and the remoulded loess) increased when the wetting-drying cycle was increased from 0 cycles to 3 cycles. For original loess, the average diameter of the particles increased from 18.27 μm to 25.43 μm with increasing moisture content before the wetting-drying cycle. Furthermore, the average diameter of the particles increased from 22.97 μm to 27.15 μm with increasing moisture content after three wetting-drying cycles. For the remoulded loess, the average diameter increased from 20.60 μm to 21.96 μm with increasing moisture content before the wetting-drying cycle. At the same time, the average diameter decreased from 27.45 μm to 25.66 μm with increasing moisture content after three wetting-drying cycles. From these results, the average diameter of remoulded loess particles exhibited a narrower range than that of original loess particles under the same wetting-drying cycle. It indicated that remoulded loess particles display a more uniform size than original loess particles. Especially after three wetting-drying cycles, the average diameter of remoulded loess particles with 15% moisture content is 25.97 μm, which is very close to 25.66 μm, the value with 25% moisture content. However, the increment of the average diameter of the original loess particles is higher than that of the remoulded loess after three cycles. It can be inferred that the size change of the original loess is more sensitive to water.

**Table 2:** The average particle diameter of different particles in original loess and remoulded loess.

Soil specimen	Moisture content	Cycles	Average Particle Diameter(μm)
Original loess	5%	0	18.27
	15%	0	20.11
	25%	0	25.43
	5%	3	22.97
	15%	3	27.97
	25%	3	27.15
Remoulded loess	5%	0	20.60
	15%	0	16.02
	25%	0	21.96
	5%	3	27.45
	15%	3	25.97
	25%	3	25.66

Compared from a perspective of moisture content, the increment in the average diameter of particles with 15% moisture content was larger than that of loess samples with other moisture content. Xu guang Xing *et al*. have reported that the particles with the initial 15% level of moisture content were prone to dissolve the salts easily on the joint between the loess particles during the wetting-drying cycle process^23^. Therefore, dissolved salts in loess recrystallized and wrapped on the surface during the wetting-drying cycle process, which led to the increasing average diameter of loess particles, especially for particles with 15% moisture content.

### The shape of loess particles

#### The ratio changes of short diameter to long diameter

**Figure 5:**
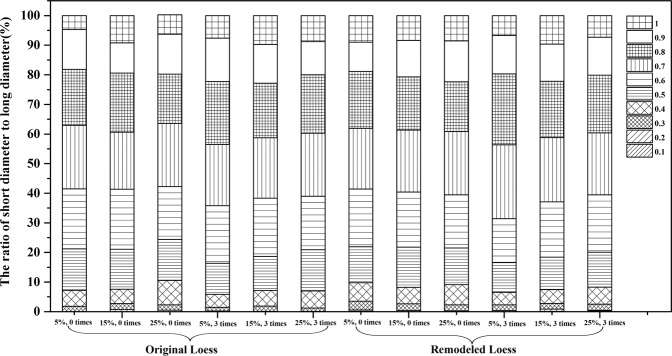
The ratio changes of short diameter and long diameter in all samples.

Using Eq. 3, the ratio of short diameter to the long diameter of the particles in all the samples was obtained, as shown in Fig. 5. The ratios of short diameter to long diameter of all samples were dominant in the range of 0.6~0.8. The closer to 1 the ratio is, the more similar to the circle shape of the particles were. The results indicated that the particles in all samples were prone to having an oval shape.

The effect of wetting-drying cycles on the ratio of the short diameter to the long diameter of the particles is similar in two kinds of loess samples. Among the original loess particles, the percentage of the values from 0.5 to 0.7 was smaller after three wetting-drying cycles, while the percentage of the values from 0.8 to 1.0 increased under the same condition. Among the remoulded loess particles, the percentage of the values from 0.5 to 0.6 was smaller after three wetting-drying cycles, while the percentage of the values from 0.7 to 1.0 increased under the same condition. This clearly illustrates that the ratio of short diameter to long diameter changed due to the wetting-drying cycles, and the short diameter measurement gradually approached that of the long diameter. This means that the shape of the loess particles altered from angular and long strips to the oval^24^. Furthermore, compared to the original loess, the remoulded loess particles more inclined to become a regular shape after the wetting-drying cycles. These results agree exceptionally well with the variations of the particles’ sizes.

#### The average shape factor of loess particles

**Figure 6:**
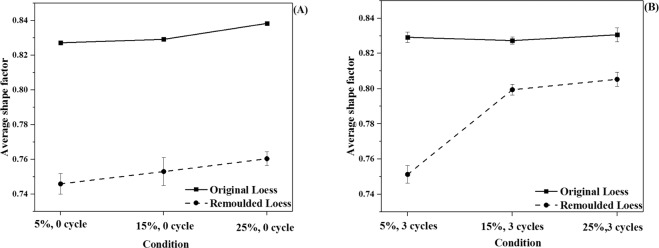
The average shape factor of loess particles. (**A**) no cycle; (**B**) three wetting-drying cycles.

Using Eq. 4, we calculated the average shape factor of the loess particles after zero and three cycle times, respectively, and the results are shown in Fig. 6. The average shape factor was used to explain the proximity of the actual particles’ surface is to the equivalent circumference. All the samples, after the same number of wetting-drying cycles, showed the average shape factor increased with the increasing of the moisture content. For 0 cycle, the average shape factor of original loess increased from 0.827 to 0.838, while the average shape factor of remoulded loess increased from 0.745 to 0.760. After three wetting-drying cycles, the average shape factor of original loess increased from 0.827 to 0.831, while the average shape factor of remoulded loess increased from 0.751 to 0.805. Before and after wetting-drying cycles, the average shape factor of remoulded loess with 15% moisture content increased most obviously among all samples, varying from 0.753 to 0.800.

Generally, before wetting-drying cycles, the average shape factor of original loess particles was higher than that of remoulded loess particles. After three wetting-drying cycles, the difference in the average shape factor of both two loess samples with 5% initial moisture content is similar to that before wetting-drying cycles. However, the average shape factor of both original loess particles and remouled loess particles with 15% initial moisture content were very close to that with 25% initial moisture content.

To further illustrate the shape changes of loess particles, we calculated the circularity of loess particles and morphological fractal dimension of loess particles by Eqs. 5 and 6. The results were displayed in Figs. S6 and S7 of the supporting document. These results are in agreement with that indicating that the wetting-drying cycles made the loess particles approach to the average shapes, especially in the case of the remoulded loess. The details were discussed in the supporting document.

### The arrangement of loess particles

#### Directional frequency of loess particles

**Figure 7:**
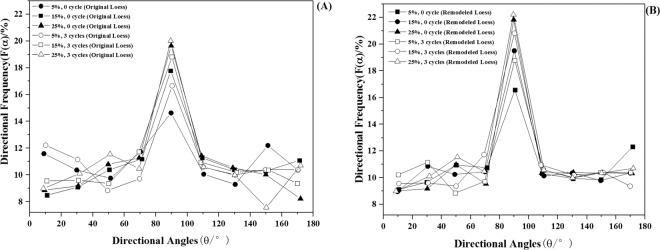
The directional frequency of loess particles before and after wetting-drying cycle: (**A**) original loess; (**B**) remoulded loess.

The directional frequency of the samples was calculated according to Eq. 7, which was used to illustrate the movement direction of loess particles under different wetting-drying cycle times^25,26^. As shown in Fig. 7, directional frequency indicated remoulded loess appeared to be more vertically aligned than original before and after three wetting-drying cycles, although numerous particles in both two loess samples fell within the range of 80°–90°. The directional frequency of all the samples increased with the increasing of moisture content. Furthermore, the directional frequency of the original loess was less than that of the remoulded loess at the same moisture levels. We can estimate that remoulded loess is more prone to move in a horizontal direction, due to its vertical compaction during the preparation process^27^. However, the particles of the original loess endured forces from different directions.

The original loess showed a similar dependence of directional frequency on moisture content after the different cycle times, and the wetting-drying cycles significantly increased the orientation frequency of the particles (Fig. 7A). Compared to the original loess, the remoulded loess appeared to be more vertically aligned (Fig. 7B). It was noticed that the directional frequency value of remoulded loess is bigger than that of the original loess in the same condition. Charles Wang Wai Ng *et al*. has reported that compacted loess (remoulded loess) exhibited more particle rearrangement than original loess under wetting-drying condition^28^. Therefore, this change in our work can be attributed to the instability of the remoulded loess particles formed during the wetting-drying cycle process.

#### The variation of directed anisotropy, directed probability entropy and directional fractal dimension of loess particles

**Figure 8:**
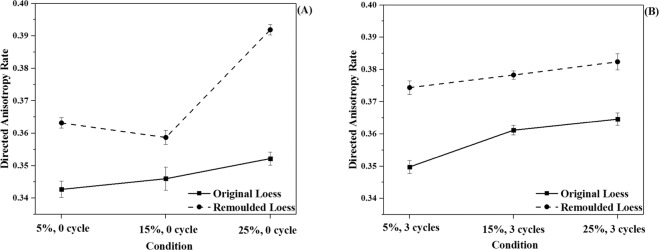
Directed anisotropy rates of loess particles before and after wetting-drying cycles: (**A**) before wetting-drying cycles; (**B**) after wetting-drying cycles.

The orientation of the loess particles plays a vital role in engineering, which is related to the sensitivity of the loess particles before and after wetting-drying cycles. The orientation of the grains in loess can be described in terms of the inclination of the particle axes to a set of reference axes^29^. In this experiment, the directed anisotropy, directed probability entropy, and directed fractal dimension of all samples were chosen as the orientational parameters. They were obtained by using Eqs. 8–10, and the results are shown in Figs. 8, S8 and S9, respectively. As shown in Fig. 8, the directed anisotropy rate (*I*_*n*_) mainly varied from 0.343 to 0.392. The directed anisotropy rate of all the samples increased with the increasing of moisture content at the same cycle time. Also, the directed anisotropy rate (*I*_*n*_) of all the samples increased after three cycles at the same moisture content levels. It was easily inferred that both the wetting-drying cycles and the increased moisture content enhanced the regular arrangement of the loess particles in various directions. At the same condition, the directed anisotropy rate of the remoulded loess was higher than that of the original loess and caused by there being more ordered particles in the remoulded loess. Both directed probability entropy and directional fractal dimension exhibited similar slight changes with the external condition. The details were demonstrated in the supporting document. It can be concluded that the arrangement of loess showed a propensity to be simple, tidy, and orderly. These results also agreed with the views reported above.

### The proposed microstructural evolution mechanism of original loess particles and remoulded loess particles during the wetting-drying cycle

From the results above, we can conclude that the size, shape, and arrangement of both original loess particles and remoulded loess particles varied in different degrees before and after three wetting-drying cycles. The microstructural comparison of the original loess and the remoulded loess suggests that the changes are dependent on the interaction between the water and loess particles during the wetting-drying cycle. Due to the effects of their initial moisture content and wetting-drying cycles, a series of complicated physical and chemical changes took place, leading to the microstructural variety in size, shape, and arrangement of the loess particles.

From these results, we found that wetting-drying cycles made all of the loess particles uniform in size, elliptical in shape, directional in the arrangement, and this applied especially to the original particles. Furthermore, there are apparent differences in the shape of the two types of loess particles before and after the wetting- drying cycles; the remoulded loess particles had variance slightly in the shape of their particles, while the original loess altered to the regular shape of particles from various fractional shape. This is mainly because the surface of remoulded loess particles was compacted to homogeneous coating by the compressing during the preparation processing, and these surfaces were kept during wetting-drying cycles. However, the various surface of original loess particles mainly formed by the reaction of calcium carbonate, clay minerals, and silts, which displayed diverse shapes on different natural environment conditions.

**Figure 9:**
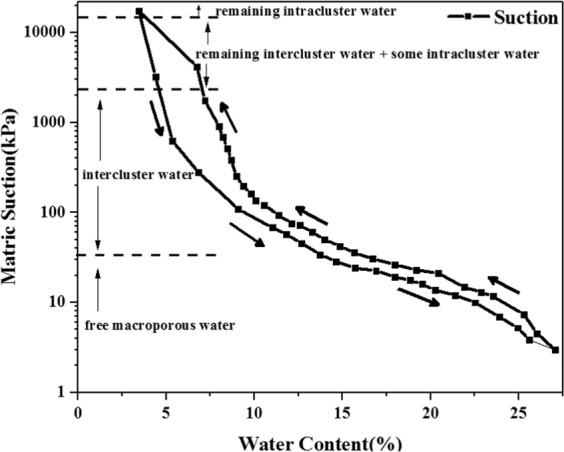
Water retention curve of the loess (‘Intra’ and ‘inter-aggregate governing suction’ zones).

In order to understand the microstructural evolution mechanism of two loess samples, the water retention curve of loess under wetting-drying cycle, shown in Fig. 9, was used to illustrate the changes of loess particles in detail^30^. The water retention curve under the wetting-drying cycle demonstrates that moisture content in samples affects the matric suction between particles. When the moisture content is low, the suction value is big and the water mainly enters the space interior of soil aggregates as intercluster water. When the moisture content is high, the suction value is small, and the water mainly enters the space between soil aggregates as free macroporous water.

**Figure 10:**
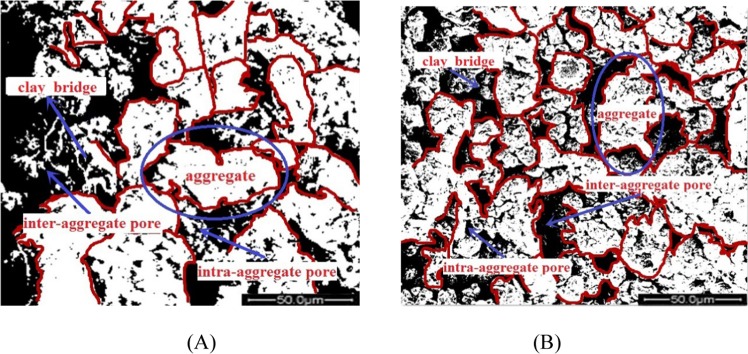
The initial analysis of binarized images for different loess particles. (**A**) the original loess at 25% moisture content with no cycle; (**B**) the remoulded loess particles at 25% moisture content with no cycle.

In general, it is well known that the microstructure of loess was considered to an open packing arrangement of pelletized aggregates of loess, which is composed of silt grains, coated by soluble salts and contacted by clay bridge. This was also proved in our binarized images shown in Fig. 10. Both two types of loess exhibited loose structure, containing aggregates, clay bridges, inter-aggregates pores, and intra- aggregates pores, resulting in a high void ratio. Among these, the clay bridge is including clay minerals and calcium carbonate, also called clay cementations or carbonate cementations. It can fix the grain in a certain place of loess. From the Ian J. Smalley’s view^31^, this clay bridge exhibited meniscus fabric separating and binding the dispersed silt grains. Before the wetting-drying cycles, both the original loess and remoulded loess displayed aggregates, clay cementations, and inter-aggregate pores. Aggregates are fundamental elements in remoulded loess, and few clean sand or silt particles can be seen. However, the size of aggregates in remoulded loess is smaller than that in original loess. At the same time, the size of pore and particles in remoulded loess is more uniform than that in original loess.

**Figure 11:**
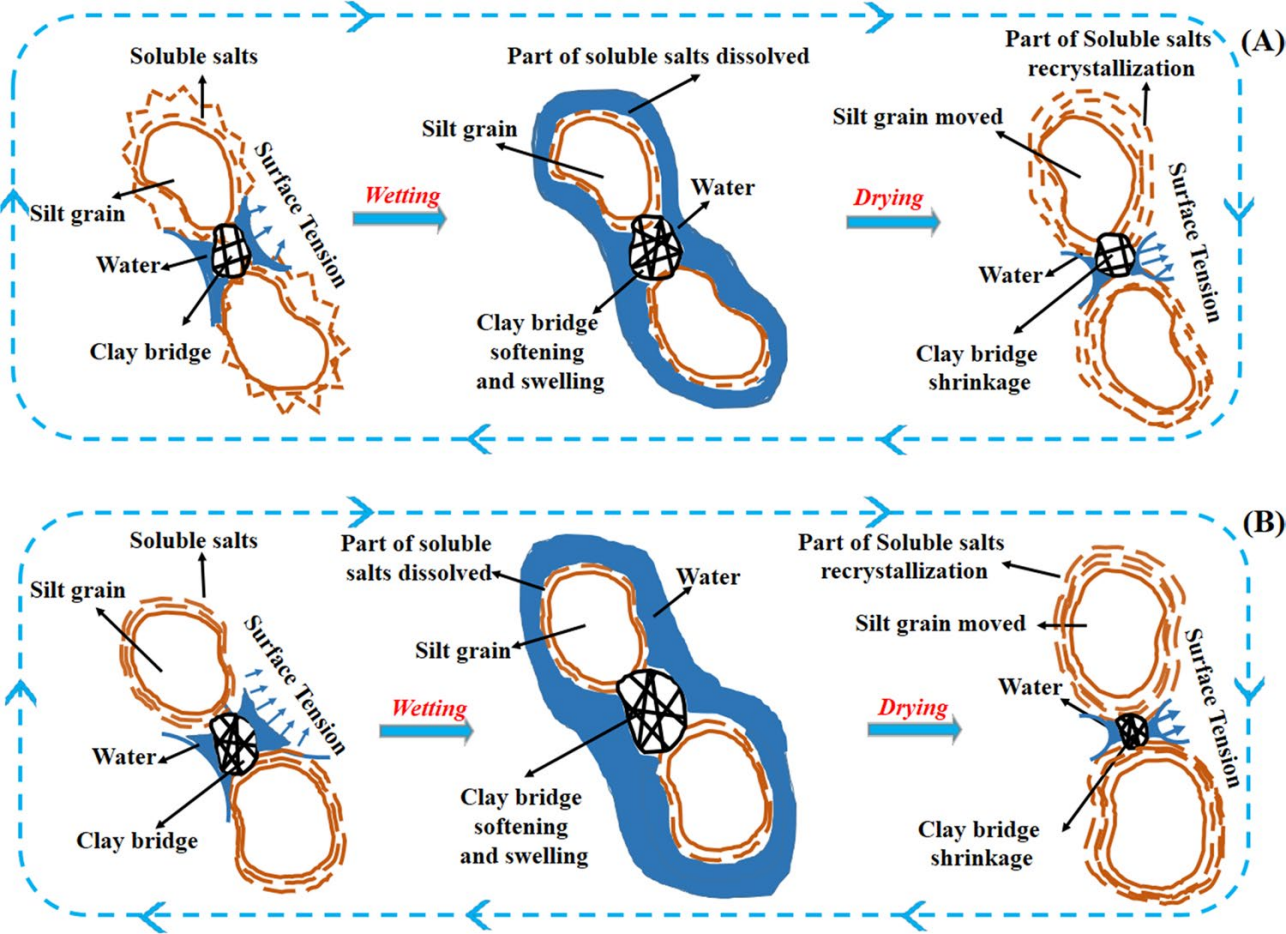
Schematic illustration of two loess particles before and after wetting-drying cycles. (**A**) original loess; (**B**) remoulded loess.

During the wetting processing, part of the fractal soluble salts coating on the surface of original loess particles was dissolved by the free macro-porous water. In addition, the clay cementations swelled and became soft, because an influx of water enters the intra-aggregate pore in clay cementations. The surface tension between adjacent silt grains, as the portion of matric suction (surface tension), was gradually reducing. On the contrary, during the drying processing, parts of dissolved salts recrystallized and coated on the surface of particles as soil water content decreased. Furthermore, the clay cementations start to uneven shrinkage. The surface tension between adjacent grains restores to a high value with the formation of the meniscus on the clay bridge, which tends to take back their water-adsorbent before wetting. As the wetting-drying cycles are made, they can repeat these steps above. Therefore, it can be inferred that dissolution and recrystallization of soluble salts coating on the surface of loess particles altered the size and shape of loess particles, and the surface tension, as parts of matric suction, accompanied by swelling-shrinking of meniscus clay cementations, rearranged the loess particles. Combined with our previous results, the particles are inclined to be fixed in a vertical or almost vertical direction. The remoulded loess also exhibited similar microstructure to original loess changes after wetting-drying cycles, especially in high moisture content. Based on these investigations, the microstructure changes of two types of loess particles due to moisture content and wetting-drying cycles are idealized in Fig. 11.

## Conclusion

Based on a morphology analysis of loess particles obtained from an SEM, the microstructure of two different types of loess samples have been quantitatively analyzed, including the results of increasing the samples’ moisture content before and after wetting-drying cycles. The results suggest that the remoulded loess exhibited similar changes in shape parameters but different variations in a directional arrangement as compared to the original loess caused by the effects of the wetting-drying cycles. Combined with the water retention curve under the wetting-drying cycle, the reason for microstructure changes of loess particles was inferred that salts effect on the size and shape of aggregates by dissolution and recrystallized in different water, clay cementations effect on the arrangement of loess particles by swelling action and suction. Furthermore, which depends on the initial moisture content and connectivity of loess particles. Compared to the remoulded loess, the original loess was more sensitive to water as a result of wetting-drying cycles.

## Supplementary information

Supplementary information.
