# Supplementary material for: Comparison and quantitative analysis of microstructure parameters between original loess and remoulded loess under different wetting-drying cycles
Source: Sci Rep. 2020 Mar 26;10:5547. doi: 10.1038/s41598-020-62571-1 (PMC7099037; doi:10.1038/s41598-020-62571-1)
Supplement: Supplementary file 1 — Supplementary information. [file 41598_2020_62571_MOESM1_ESM.doc]

**Comparison and quantitative analysis of microstructure parameters** **between original loess and remoulded loess under different** **wetting-drying cycles**

Wan-kui Ni^a*^, Kang-ze Yuan^a^, Xiang-fei Lü^a,b*^, Zhi-hui Yuan

^a^*Department of Geological Engineering,* *College of Geological Engineering and Surveying and Mapping, Chang’an University, No.126 Yanta Road, Xi'an, Shaanxi 710054, P.R. China*

^b^*School of Environmental Science and Engineering, Chang’an University, No. 126 Yanta Road, Xi’an, Shaanxi 710054, P. R. China*

*^c^College of Water Conservancy and Ecological Engineering, Nan Chang Institute of Technology, Nan Chang, Jiangxi 330099, P.R. China*

^*^*Corresponding author.* *E-mail addresses:* [*nnwwkk@126.com*](mailto:junli@nwu.edu.cn)*; Sophie_Lv@126.com*

*tel: +0086 29 82334141, fax: +0086 29 82334137*

**Supplementary information**

***1. Morphology of all samples***

Cubic sticks, having the dimensions of approximately 1 cm × 1 cm × 2 cm (length × width × height, were trimmed out from the central part of the loess samples after the drying-wetting cycles. Before scanning, the soil sticks were slightly fractured by hand at about 1 cm height, and the new surface was used to examine the microstructure of the samples. Figure S1 shows the surface of the samples by the SEM. The typical morphology of loess samples under different wetting and drying cycles were presented as follows: from Figure S2 to Figure S5. In order to observe the particles clearly, all the photos were chosen in magnified 1000 times.


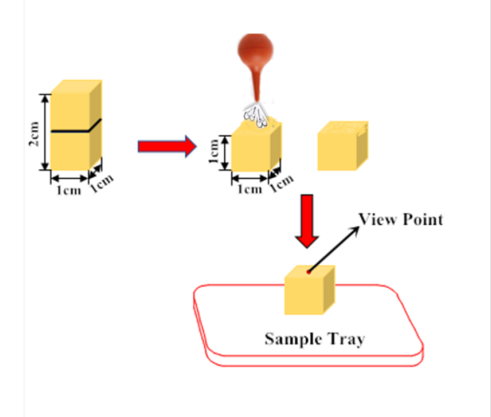


Figure S1**.** Observed surface in samples.


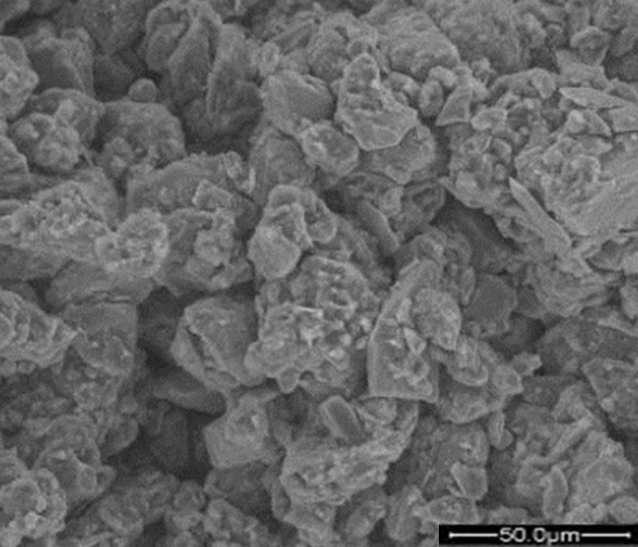

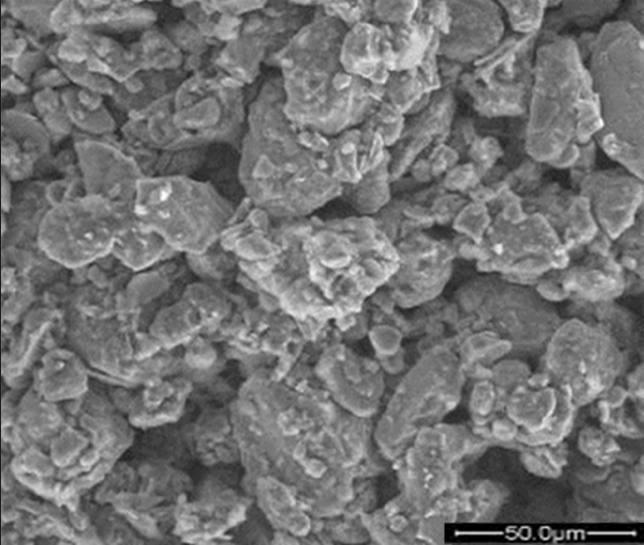

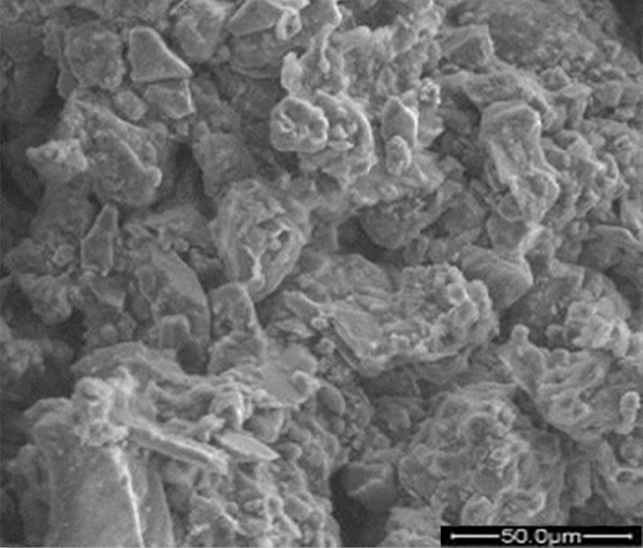


| **Figure S2.** SEM images of original loess under no cycles (The moisture contents of samples were 5%, 15%，25% from left to right, respectively.).  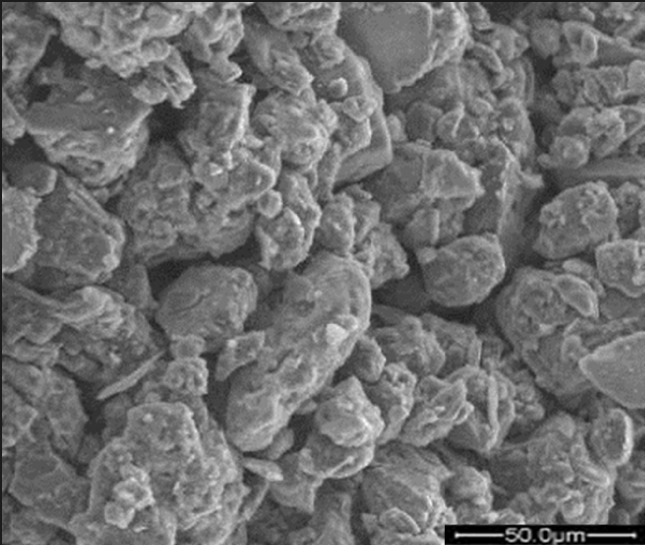 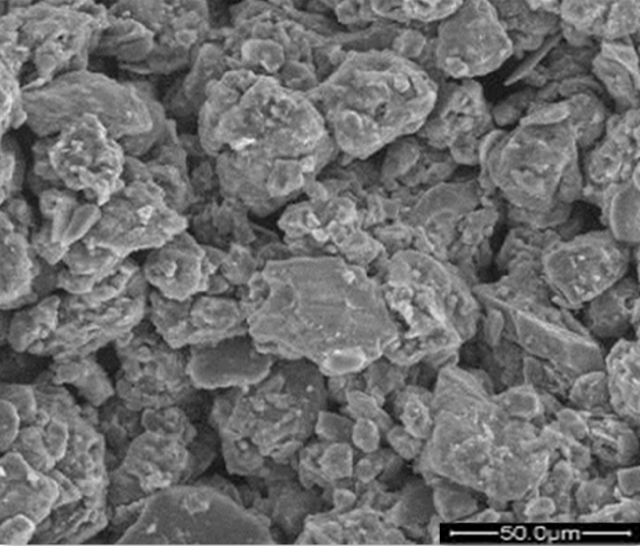 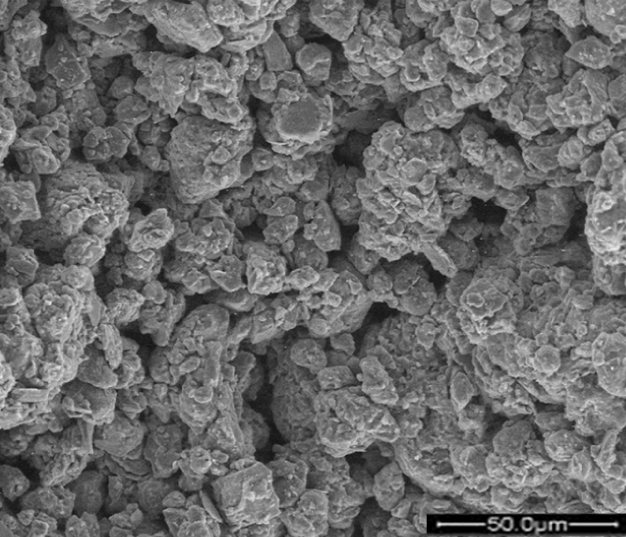 |  |
| --- | --- |
| **Figure S3.** SEM images of remodeled loess under no cycles (The moisture contents of samples were 5%, 15%, 25% from left to right, respectively.).  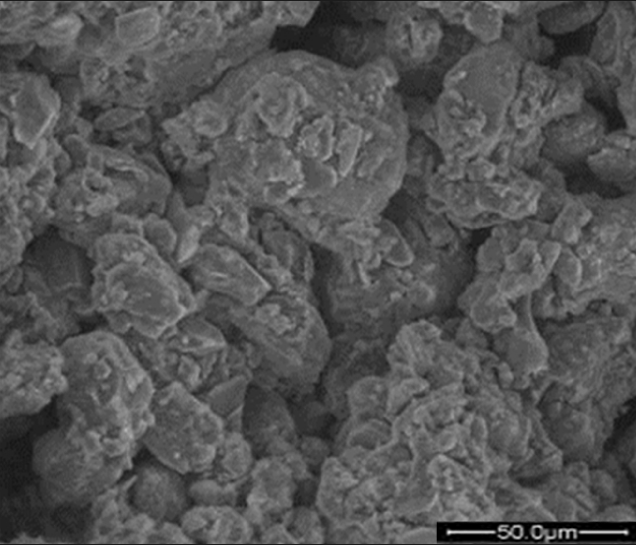 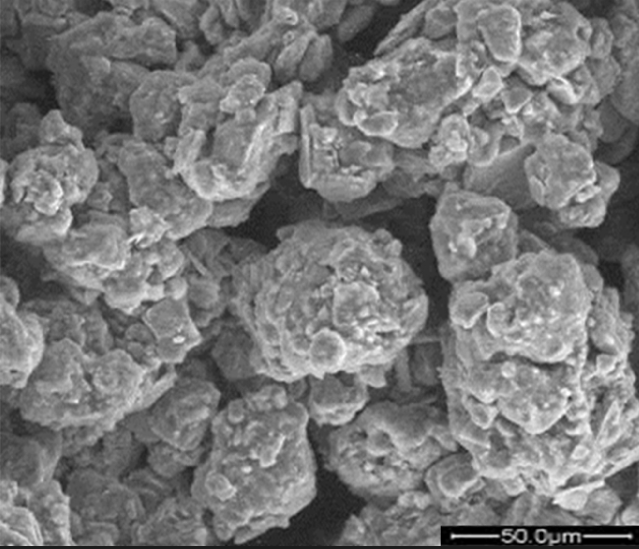 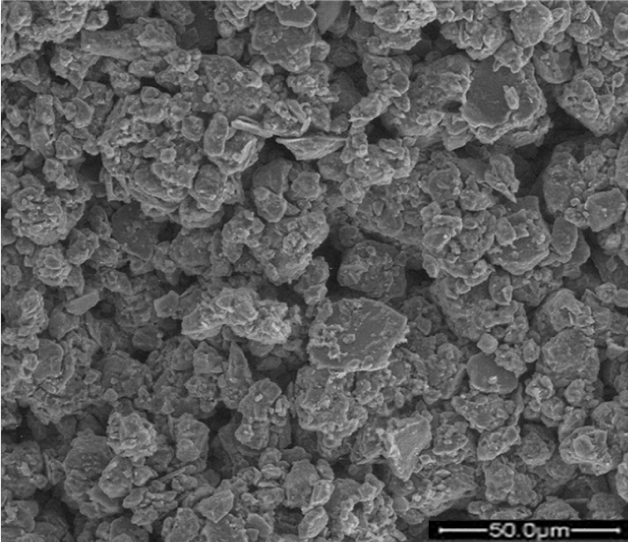 |  |
| **Figure S4.** SEM images of original loess samples after three wetting-drying cycles (The moisture contents of samples were 5%, 15%，25% from left to right, respectively.).  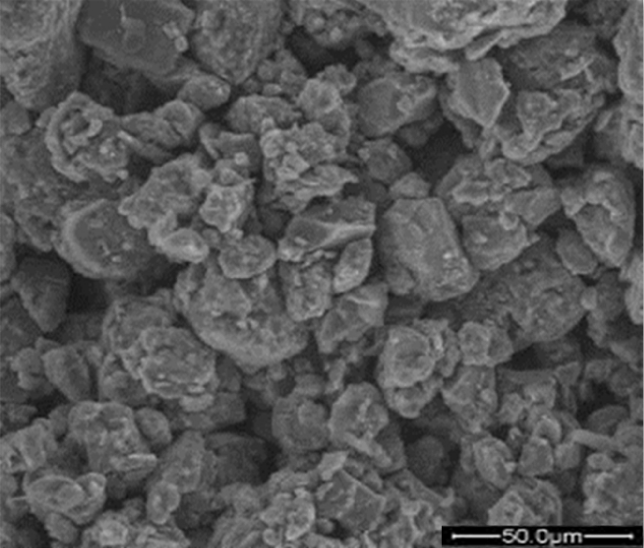 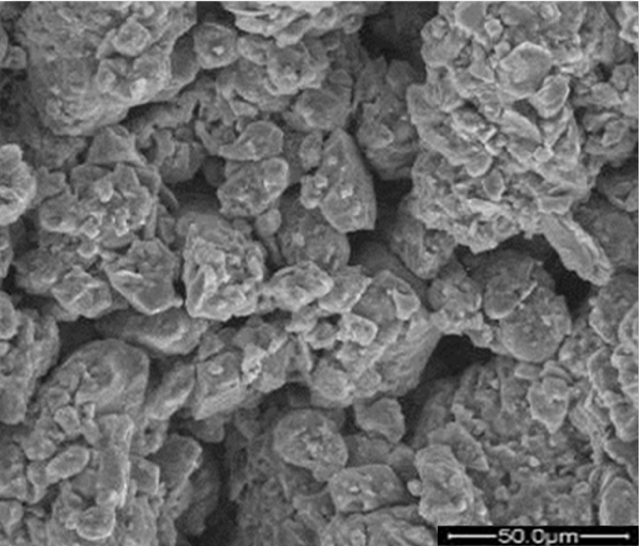 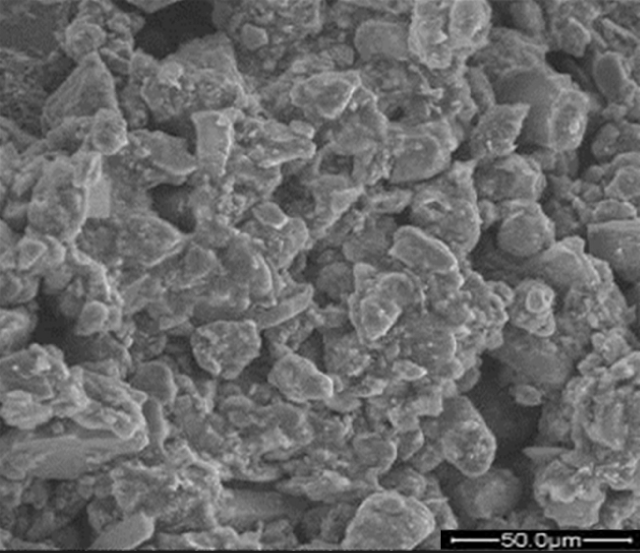 |  |
| **Figure S5.** SEM images of remodeled loess samples under wetting-drying three cycles (The moisture contents of samples were 5%, 15%，25% from left to right, respectively.). | |

***2. Method***

In order to obtained microstructure parameters, IPP 6.0 system was used to quantitative analysis of loess microstructure image. According to the literature, average diameter ***d***, the ratio of short diameter and long diameter ***C*,** circularity ***R***, the average shape factor ***F***, morphological Fractal Dimension ***D***, Directional frequency ***F(α)***，anisotropy rate ***I_n_***, directional probability entropy ***H_m_***, directional fractal dimension ***D_f_*** were chosen and calculated from the IPP 6.0 system, as the microstructural parameters of loess particles^1,2^.

***3. Results and discussion***

*3.1 The shape parameters of loess*

*3.1.1 The Circularity of loess particles*

According to equation 5, we calculated the circularity of loess particles, shown in Figure S6. Under the same conditions, the original loess particles’ circularity was likely to fall more than the circularity of the remoulded loess particles. In the beginning, (no cycle), as compared to the original loess, the remoulded loess particles possessed greater circularity, due to the destroyed connectivity of the adjacent loess particles during the compaction process. However, this advantage disappeared after the drying and wetting cycles, and the circularity became close to the same value as the original loess’s. The water dissolved the salts on the surface of the loess particles and made the particles close to a round shape during the drying and wetting cycle^3^.


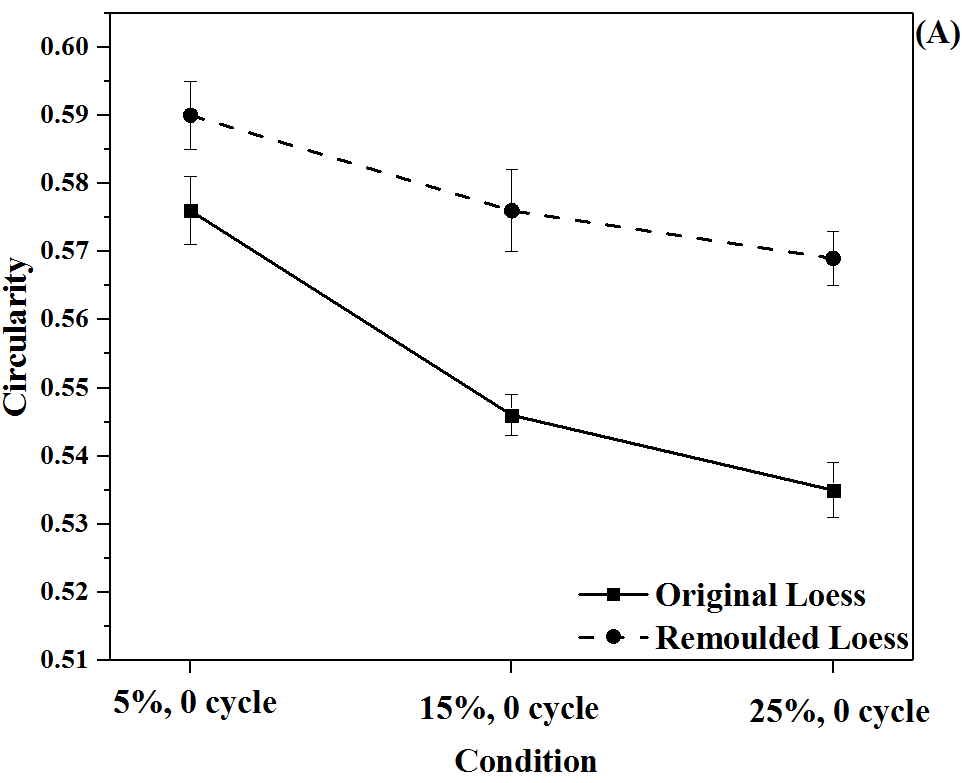

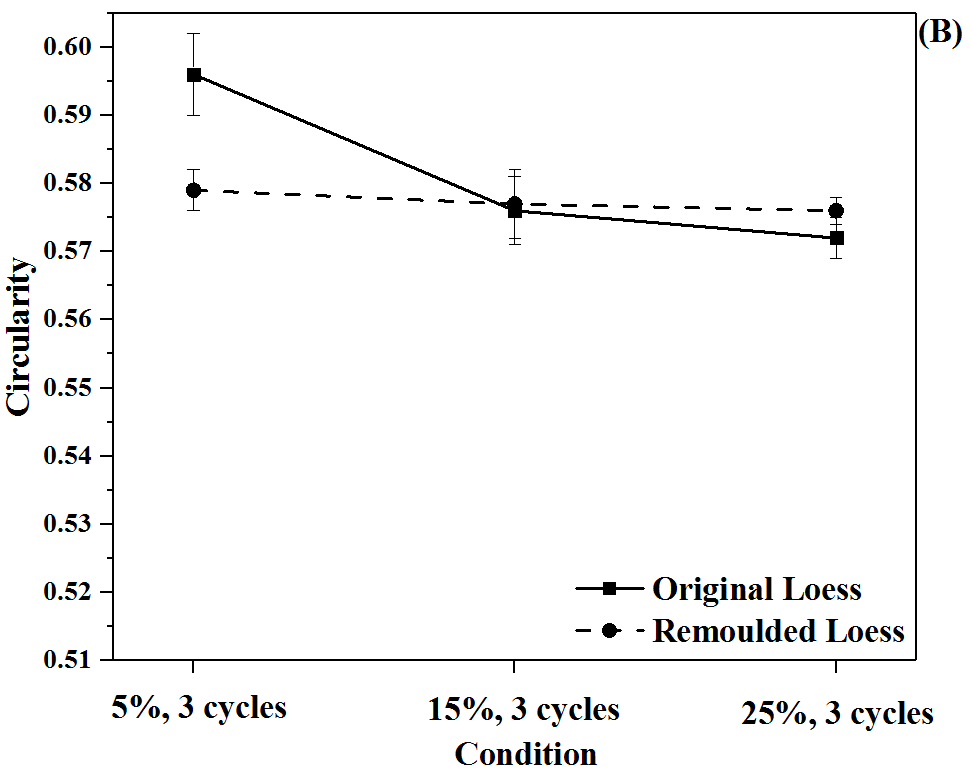


Figure S6**.** The circularity of loess particles. (A): no cycle; (B): three wetting-drying cycles.

*3.4 Morphological fractal dimension of loess particles*

The results of equation 6, the morphological fractal dimension values of all samples, are depicted in Figure S7. The results show that with increasing levels of initial moisture content, the morphological fractal dimension values of the original loess and the remoulded loess continually increased and reached the same value. After three drying and wetting cycles, with increasing initial moisture content, both the original loess and remoulded loess also increased by the same amount. Furthermore, compared to the remoulded loess particles, the morphological fractal dimension of the original loess particles was more significant. Also, the morphological fractal dimension of the original loess particles was similar before and after the drying and wetting cycles. However, in the remoulded loess particles, the morphological fractal dimension after the drying and wetting cycles was smaller than before the drying and wetting cycle. The increase of the morphological fractal dimension indicated that the surface undulation of the particles became larger and that the surface was rougher. From these results, we concluded that with increased initial moisture content, the surface of all the samples became bigger and rougher, which resulted from the smaller particles attached to the surfaces of the larger ones. However, after three drying and wetting cycles, the friction between water and particle decreased the surface roughness of the loess and dissolved the salts on the junctions between the particles. It made the loess particles disintegrate into small particles, especially the remoulded particles, which was consistent with the changes in the circularity and the average shape factor.


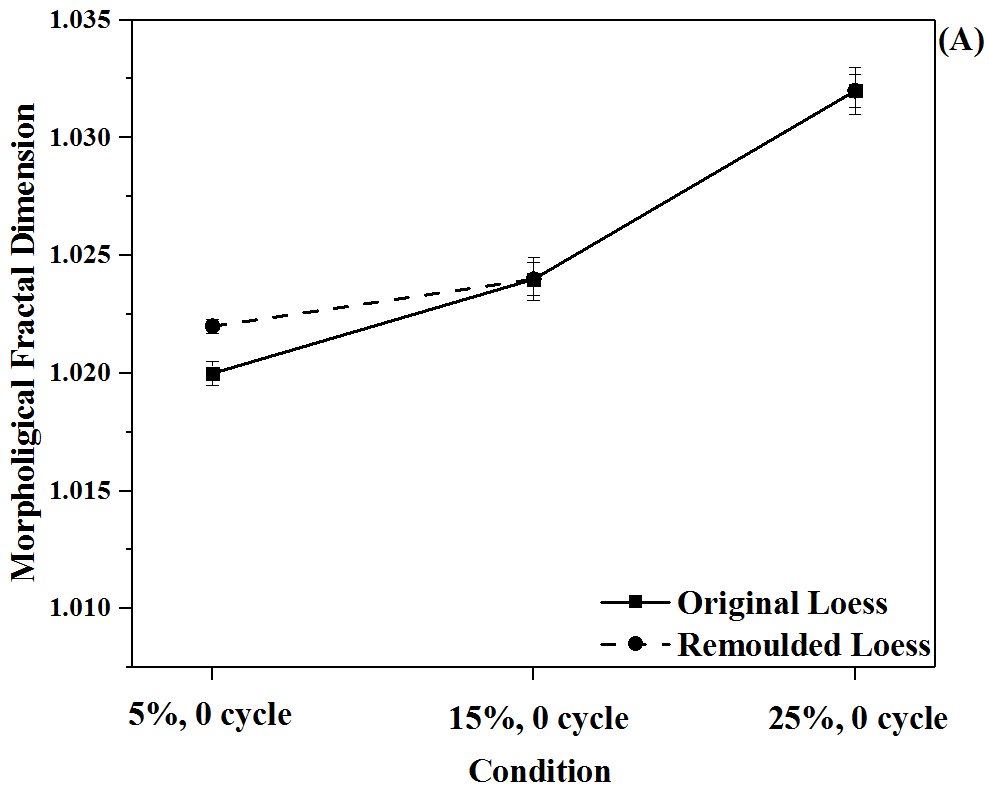

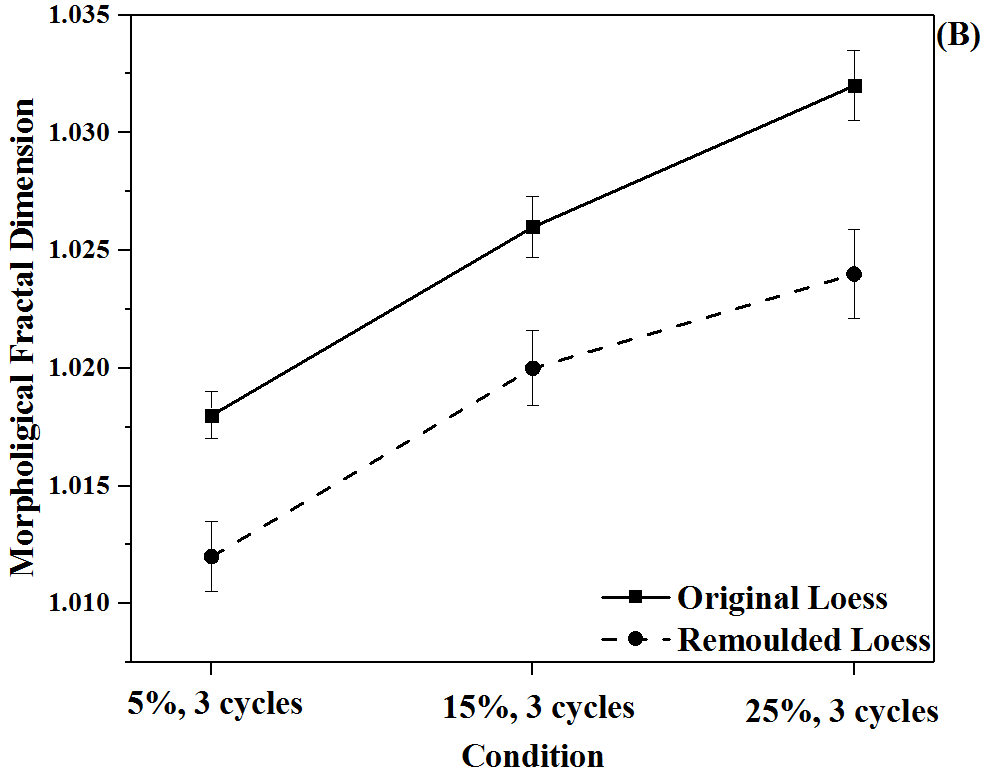


Figure S7**.** The morphological fractal dimension of loess particles. (A): no cycle; (B): three wetting-drying cycles.

*3.2 The arrangement of loess particles*

*3.2.1 The directed probability entropy*

By using Eq. 9, we calculated the directional probability entropy of loess particles, shown in Figure S8. According to the literature^4^, the directional probability entropy of loess particles is smaller, and their directionality is more consistent. With increasing moisture content, both the directional probability entropy of the original loess particles and that of the remoulded particles decreased and reached the same value. Furthermore, under the same condition, the directional probability entropy of the original loess was higher than that of the remoulded loess particles. After three drying and wetting cycles, the directional probability entropy of original loess particles decreased slightly. These results demonstrated that the arrangement of the soil particles in the original loess is relatively complicated and disorderly, and the orientation of the original loess is worse than that of remoulded loess^5^. The drying and wetting cycles arranged the loess particles in various directions. This effect is more evident in the remoulded loess. According to a previous work^6^, this can be attributed to the arrangement of and contact with unstable soil particles altered by their dissolution in water during the drying and wetting cycle process, which obliges the particles to balance their force in various directions.


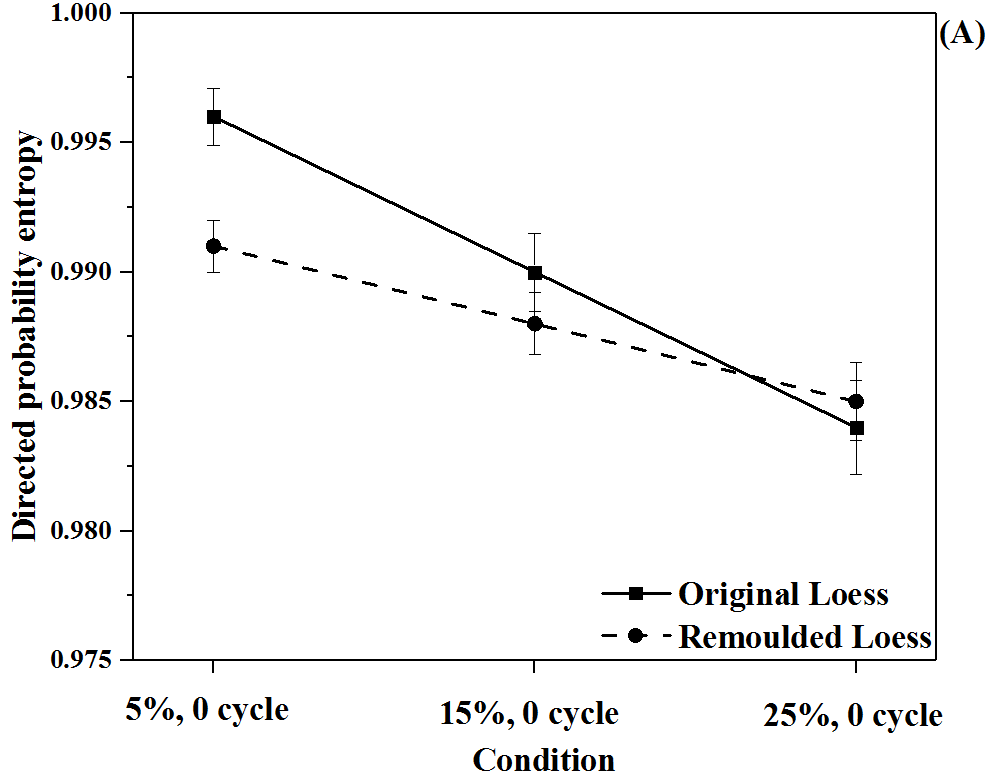

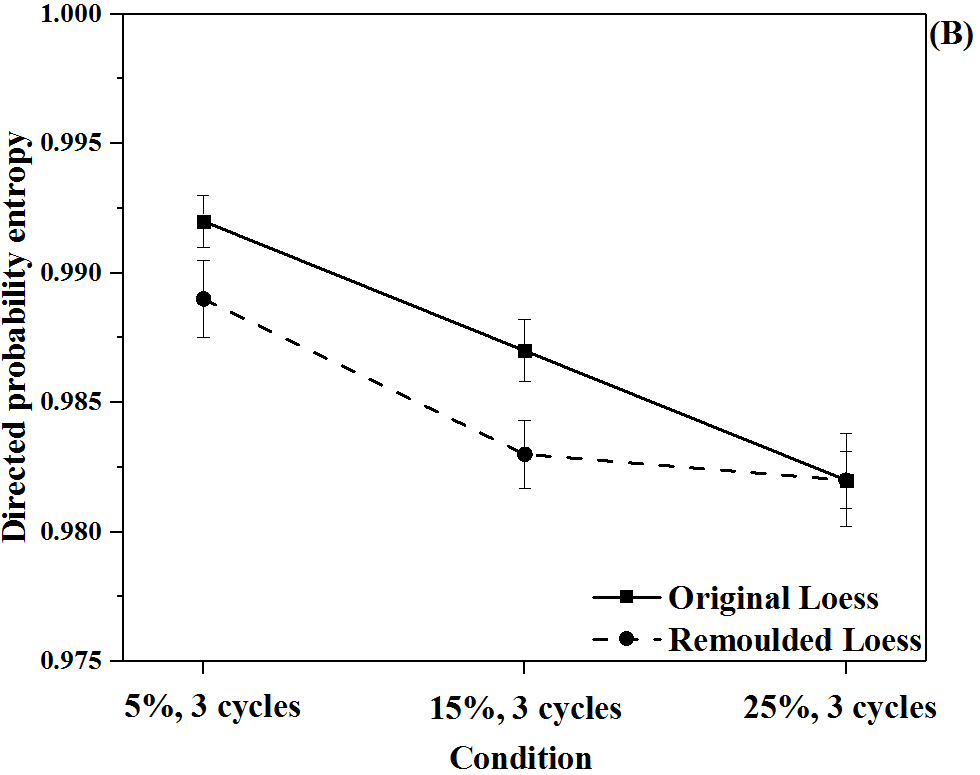


Figure S8**.** The directed probability entropy of loess particles before and after wetting-drying cycles : (A): before wetting-drying cycles; (B): after wetting-drying cycles.

3.2.2 *The directional fractal dimension of loess particles*

By using Eq. 10, we obtained the directional fractal dimension of the loess particles, displayed in Figure S9. The directional fractal dimension of the loess particles decreased with increased moisture content. It can be concluded that the arrangement of loess showed a propensity to be simple, tidy, and orderly.


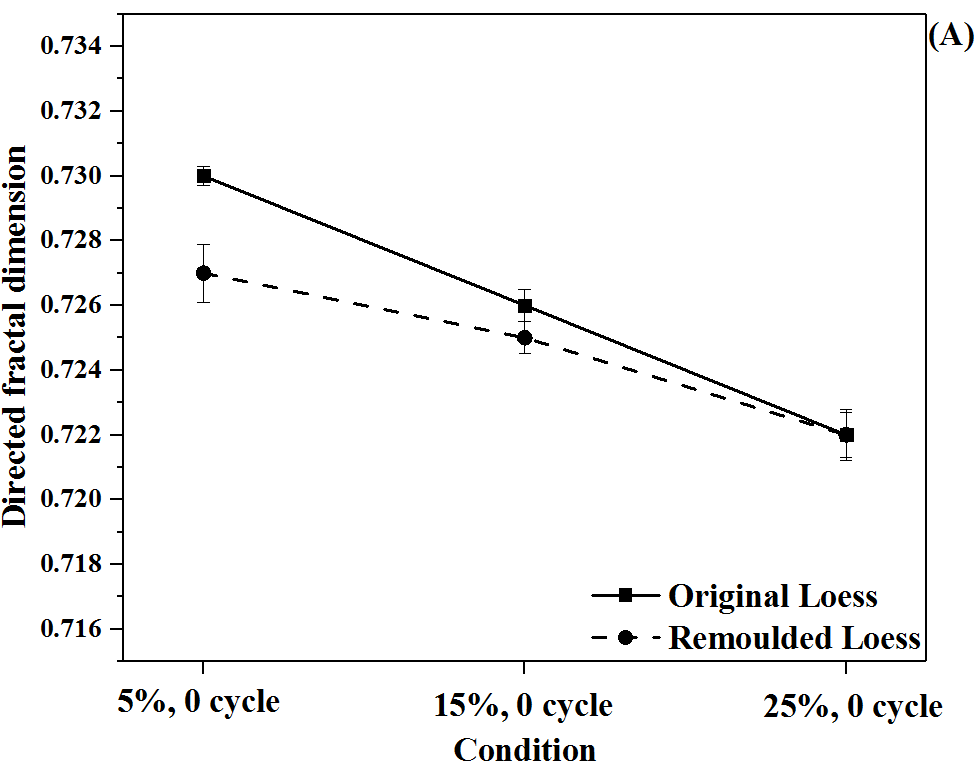

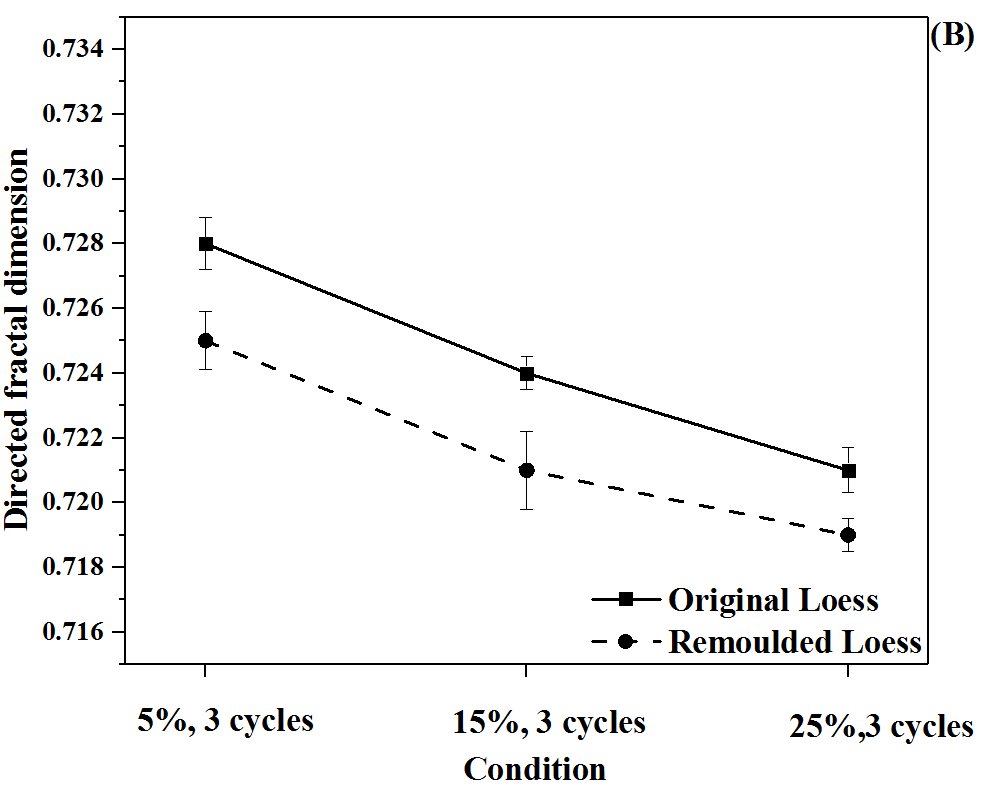


Figure S9**.** The directed fractal dimension of loess particles before and wetting-drying cycles: (A): before wetting-drying cycles; (B): after wetting-drying cycles.

**Reference:**

1. Xie, X., Qi, S., Zhao, F. & Wang, D. Creep behavior and the microstructural evolution of loess-like soil from Xi’an area, China. Engineering Geology 236, 43–59 (2018).
2. Zhenguo, Y. et al. Characteristics of limestone manufactured sand. 6
3. MUñOZ-CASTELBLANCO, J. A., et al. The water retention properties of a natural unsaturated loess from northern France. Géotechnique 62, 95–106 (2012).
4. Haeri, S. M., et al. Effect of Soil Structure and Disturbance on Hydromechanical Behavior of Collapsible Loessial Soils. Int. J. Geomech. 17, 04016021 (2017).
5. Luo, H., et al. Microstructural constraints on geotechnical properties of Malan Loess: A case study from Zhaojiaan landslide in Shaanxi province, China. Engineering Geology 236, 60–69 (2018).
6. Otalvaro, I. F., et al. Relationship between soil structure and water retention properties in a residual compacted soil. Engineering Geology 205, 73–80 (2016).
